# Effects of elastic-band resistance training on glycemic control, muscle strength, and functional outcomes in patients with type 2 diabetes mellitus: a systematic review and meta-analysis of randomized controlled trials

**DOI:** 10.3389/fpubh.2026.1910792

**Published:** 2026-08-21

**Authors:** Yongliang Zhu, Zhaoying Zhu, Haozhe Wang, Sicheng Cao, Changkun Shan, Xiaotong Yuan

**Affiliations:** 1 School of Physical Education, Shandong Sport University, Jinan, China; 2 School of Sports Medicine and Rehabilitation, Beijing Sport University, Beijing, China; 3 School of Physical Education, China University of Mining and Technology, Xuzhou, China; 4 Department of Public Health, Jinan Vocational College of Nursing, Jinan, China

**Keywords:** elastic band, glycemic control, handgrip strength, meta-analysis, physical function, randomized controlled trial, resistance training, type 2 diabetes mellitus

## Abstract

**Background:**

Patients with type 2 diabetes mellitus (T2DM) commonly experience reduced muscle strength and impaired physical function, which may complicate long-term disease management. Elastic-band resistance training is inexpensive, portable, and suitable for home-based exercise; however, evidence regarding its effects on glycemic control, muscle strength, physical function, and muscle mass has not been comprehensively synthesized. This systematic review and meta-analysis evaluated the effects of elastic-band resistance training on these outcomes in adults with T2DM.

**Methods:**

PubMed, Web of Science, China National Knowledge Infrastructure (CNKI), Wanfang Data, and VIP Database were searched from inception to June 5, 2026 for randomized controlled trials (RCTs) in adults with T2DM. Intervention effects were summarized using mean differences (MDs) or standardized mean differences (SMDs), and certainty of evidence was assessed using GRADE.

**Results:**

Fourteen RCTs involving 684 participants were included. Elastic-band resistance training was associated with reductions in HbA1c (MD = −0.41, 95% CI −0.62 to −0.20) and fasting plasma glucose (FPG; MD = −0.79, 95% CI −1.01 to −0.56), with moderate-certainty evidence for both outcomes. Leave-one-out sensitivity analyses yielded consistent results, supporting the robustness of the primary findings. Handgrip strength also improved (MD = 2.67, 95% CI 1.07 to 4.26; I^2^ = 0%), with moderate-certainty evidence. Muscle-mass outcomes favored elastic-band resistance training (SMD = 0.24), although the confidence interval approached the null value and the certainty of evidence was low. Walking ability and sit-to-stand performance also showed favorable trends; however, substantial heterogeneity reduced confidence in these estimates. Adverse events were inconsistently reported, precluding firm conclusions regarding intervention safety.

**Conclusion:**

Elastic-band resistance training may improve glycemic control and muscle strength in adults with T2DM, whereas its effects on walking ability, sit-to-stand performance, and muscle mass remain uncertain. Given its low cost, portability, and suitability for home-based exercise, it may represent a practical adjunct to comprehensive exercise management, particularly for individuals with limited access to conventional resistance-training equipment. Safety remains insufficiently characterized because adverse-event reporting was incomplete. Further high-quality randomized controlled trials are warranted to strengthen the evidence base and refine optimal training prescriptions.

**Systematic review registration:**

www.crd.york.ac.uk/prospero, CRD420261417541.

## 1 Introduction

Type 2 diabetes mellitus (T2DM) is a metabolic disorder characterized primarily by chronic hyperglycemia and insulin resistance. Its prevalence continues to increase, and long-term disease management remains a major challenge in public health and clinical practice (1). Glycated hemoglobin (HbA1c) and fasting plasma glucose (FPG) are commonly used indicators of glycemic control in both clinical care and research. They reflect overall and basal glycemic status, respectively, and should therefore both be considered when evaluating the effects of exercise interventions (2). In addition to dysglycemia, patients with T2DM frequently experience reduced skeletal muscle mass, diminished muscle strength, and impaired physical function. Previous studies have reported an association between T2DM and sarcopenia, suggesting that individuals with T2DM may be at greater risk of muscle loss and weakness than those without diabetes (3, 4). Because skeletal muscle is the primary site of glucose uptake and utilization and plays a central role in mobility and activities of daily living, management of T2DM should extend beyond glycemic control to include the preservation of muscle health and physical function.

Exercise is a cornerstone of comprehensive T2DM management. Previous systematic reviews have shown that structured exercise, including aerobic exercise, resistance training, and combined exercise, may improve glycemic control (5). The American Diabetes Association also recommends resistance training as an important component of exercise prescriptions for individuals with T2DM because of its potential benefits for glycemic management and overall health (2). Compared with exercise programs focused primarily on aerobic capacity, resistance training may provide additional benefits for improving muscle strength, preserving lean tissue, and maintaining physical function, particularly in older adults and individuals with functional limitations (6). However, conventional resistance training often requires specialized equipment, fixed machines, or free weights, which may limit accessibility and long-term adherence. Elastic-band resistance training is portable, inexpensive, easily adjustable, and suitable for home-based implementation. Training intensity can be modified through band resistance, elongation, and exercise selection, making it a practical alternative to conventional resistance training, particularly for individuals with limited access to supervised exercise facilities or specialized equipment.

Previous evidence has suggested potential benefits of elastic-band resistance training; however, important knowledge gaps remain. A systematic review of elastic resistance training in older adults reported improvements in muscle strength and functional performance, although the magnitude of benefit varied across populations and outcomes (7). An earlier meta-analysis in patients with T2DM provided a preliminary synthesis of glycemic control and muscle strength but included relatively few studies and did not comprehensively evaluate physical function or muscle-mass outcomes (8). Since the publication of that review, several additional randomized controlled trials (RCTs) have become available, substantially expanding the evidence base. These studies have extended the evaluation of elastic-band resistance training beyond glycemic control and muscle strength to include clinically relevant functional and muscle-mass outcomes, highlighting the need for an updated and more comprehensive synthesis of the available evidence.

Accordingly, this systematic review and meta-analysis was conducted to evaluate the effects of elastic-band resistance training on glycemic control, muscle strength, physical function, and muscle mass in adults with T2DM. HbA1c and FPG were prespecified as the primary outcomes. Secondary outcomes included walking ability, handgrip strength, sit-to-stand performance, and muscle-mass outcomes to provide a more comprehensive evaluation of the potential benefits of elastic-band resistance training in this population.

## 2 Materials and methods

The review protocol was registered with PROSPERO (CRD420261417541). The registration specified the study objectives, eligibility criteria, interventions, comparators, and planned primary and secondary outcomes. The review was conducted in accordance with the prespecified protocol, with no major deviations, and was reported according to the Preferred Reporting Items for Systematic Reviews and Meta-Analyses (PRISMA 2020) statement (9).

### 2.1 Search strategy

We systematically searched PubMed, Web of Science, China National Knowledge Infrastructure (CNKI), Wanfang Data, and VIP Database from inception to June 5, 2026. The search strategy combined controlled vocabulary and free-text terms and was adapted to the indexing system and search syntax of each database. The search strategy comprised three concepts: (1) type 2 diabetes mellitus; (2) elastic-band resistance training; and (3) randomized controlled trials. The elastic-resistance search block included the terms “elastic band,” “resistance band,” “Thera-Band,” “elastic tubing,” “elastic resistance,” “elastic resistance exercise,” “elastic tubing exercise,” and “home resistance exercise.” Search terms were informed by terminology used in eligible trials and previous systematic reviews. Reference lists of the included studies and relevant systematic reviews were manually screened to identify additional eligible studies. The complete search strategies for all databases are provided in (Supplementary Table S1; Table 1).

**Table 1:** Database search strategy.

Search combination	Search term	Search field
#1	“Diabetes Mellitus, Type 2″	MeSH Terms
#2	“type 2 diabetes” OR “type 2 diabetes mellitus” OR T2DM OR “non-insulin-dependent diabetes” OR “diabetes mellitus type 2”	Title/Abstract
#3	“Resistance Training” OR “Exercise Therapy” OR “Elastic Bandages”	MeSH Terms
#4	“elastic band” OR “elastic bands” OR “resistance band” OR “resistance bands” OR “Thera-Band” OR Theraband OR “elastic tubing” OR “elastic resistance” OR “rubber band” OR “progressive elastic resistance” OR “elastic resistance training” OR “elastic band resistance exercise” OR “elastic resistance exercise” OR “elastic tubing exercise” OR “home resistance exercise”	Title/Abstract
#5	“Randomized Controlled Trial” OR “Controlled Clinical Trial”	Publication Type
#6	randomized OR randomised OR randomly OR “random allocation” OR “controlled trial” OR “clinical trial” OR RCT	Title/Abstract
#7	(#1 OR #2) AND (#3 OR #4) AND (#5 OR #6)	Combined

### 2.2 Eligibility criteria

Study selection was performed independently by two reviewers. Disagreements were resolved through discussion or consultation with a third reviewer. Eligibility criteria were defined using the PICO-S framework. (1) Participants: adults with a confirmed diagnosis of T2DM, with no restrictions on sex. Studies involving mixed populations were eligible only if outcome data for participants with T2DM could be extracted separately. (2) Intervention: resistance training delivered primarily using elastic bands, resistance bands, Thera-Band, elastic tubing, or other elastic-resistance devices. Elastic-band resistance training had to constitute the primary intervention rather than an auxiliary component used only for warm-up, stretching, or stabilization. For mixed interventions, studies were included only when elastic-band resistance training was the principal intervention or when co-interventions were administered equally in both groups. Studies in which the independent effect of elastic-band resistance training could not be distinguished were excluded. (3) Comparator: usual care, health education, no exercise, a waiting-list condition, maintenance of usual daily activities, conventional rehabilitation, or another active exercise intervention. (4) Outcomes: studies reporting at least one extractable outcome related to glycemic control, muscle strength, physical function, or body composition. The primary outcomes were HbA1c and FPG. Secondary outcomes included walking outcomes, muscle-mass outcomes, handgrip strength, and sit-to-stand outcomes. (5) Study design: randomized controlled trials (RCTs), including individual parallel-group randomization and other randomized designs providing valid comparative data. The exclusion criteria were as follows: (1) nonrandomized studies, observational studies, case reports, case series, reviews, meta-analyses, study protocols, conference abstracts, or reports with incomplete data; (2) studies involving type 1 diabetes, gestational diabetes, prediabetes, or healthy participants when data for individuals with T2DM could not be extracted separately; (3) interventions in which elastic-band resistance training was not the primary intervention, including aerobic exercise alone, machine-based resistance training, free-weight training, body-weight exercise, yoga, stretching, or balance training; (4) studies using elastic bands solely as an auxiliary training tool; (5) missing or insufficient outcome data that could not be converted or obtained from the study authors; (6) duplicate publications or overlapping study populations, in which case the report with the largest sample size, most complete dataset, or longest follow-up was retained; and (7) studies reporting only acute exercise responses rather than repeated exercise interventions.

### 2.3 Study selection and data extraction

Two reviewers independently selected studies and extracted data. Titles and abstracts were screened first, followed by full-text assessment. Disagreements were resolved through discussion or consultation with a third reviewer. A prespecified extraction form was used to collect: (1) study characteristics, including first author, publication year, study design, participant characteristics, sample sizes of the experimental and control groups included in the meta-analysis, age, and sex; (2) intervention characteristics, including the elastic-band training protocol, comparator, intervention duration, training frequency, session duration, and intensity. For multi-arm trials, only the elastic-band group and the comparator that met the review’s PICO criteria were extracted. Combined interventions or other exercise modalities were included in quantitative synthesis only when they met the prespecified criteria; and (3) outcome data for glycemic control, muscle strength, muscle mass, and physical function. The primary outcomes were HbA1c and FPG. Secondary outcomes included walking outcomes, muscle-mass outcomes, handgrip strength, and sit-to-stand outcomes. When multiple timepoints were reported, data collected immediately after the intervention were prioritized. For incomplete datasets, we attempted to contact the original authors; when data remained unavailable, values were converted or estimated using methods recommended in the Cochrane Handbook.

### 2.4 Risk of bias and certainty of evidence

The Cochrane Risk of Bias 2 tool (RoB 2) was used to assess the methodological quality of the included RCTs (10). The assessment covered five domains: bias arising from the randomization process, bias due to deviations from intended interventions, bias due to missing outcome data, bias in outcome measurement, and bias in selection of the reported result. Each domain was rated as “low risk,” “some concerns,” or “high risk,” followed by an overall risk-of-bias judgment for each study. The certainty of evidence was assessed using the Grading of Recommendations Assessment, Development and Evaluation (GRADE) approach (11, 12). Evidence was rated as high, moderate, low, or very low after consideration of risk of bias, inconsistency, indirectness, imprecision, and publication bias. Two reviewers completed the assessments independently. Disagreements were resolved by consensus or consultation with a third expert.

### 2.5 Statistical analysis

Meta-analyses and graphical outputs were produced using R software. For continuous outcomes, effect estimates were expressed as mean differences (MDs) or standardized mean differences (SMDs) with 95% confidence intervals (CIs). MDs were used for outcomes reported in consistent units, such as HbA1c and FPG, whereas SMDs were used when studies measured the same construct with different instruments or units. Outcome directions were harmonized before data synthesis and plotting. Statistical heterogeneity was assessed using the chi-square test and I^2^ statistic. Model choice considered both clinical/methodological diversity and statistical heterogeneity rather than I^2^ alone. Random-effects models were used when clinically meaningful between-study variation was expected or when the chi-square test indicated heterogeneity at *p* ≤ 0.10; fixed-effect models were reserved for outcomes with sufficiently comparable study characteristics and low statistical heterogeneity. To explore potential sources of heterogeneity, we prespecified subgroup analyses according to training frequency, population characteristics, and comparator type (13). Leave-one-out sensitivity analyses were performed to identify influential studies and to assess whether the direction and magnitude of the pooled estimates were robust to individual studies; these analyses were not used to select a preferred lower-heterogeneity or more statistically significant result.

Because participant characteristics, programme duration and intensity, comparator conditions, and outcome measurements varied across trials, random-effects estimates were interpreted as average effects across clinically diverse settings. Sensitivity analyses were used solely to assess robustness and were not treated as primary evidence.

When at least 10 studies contributed to an outcome, funnel plots and Egger’s test were used to assess potential publication bias. Formal testing was not performed for outcomes with fewer than 10 studies; publication bias was instead considered qualitatively when interpreting the findings (14).

## 3 Results

### 3.1 Study selection

**Figure 1:**
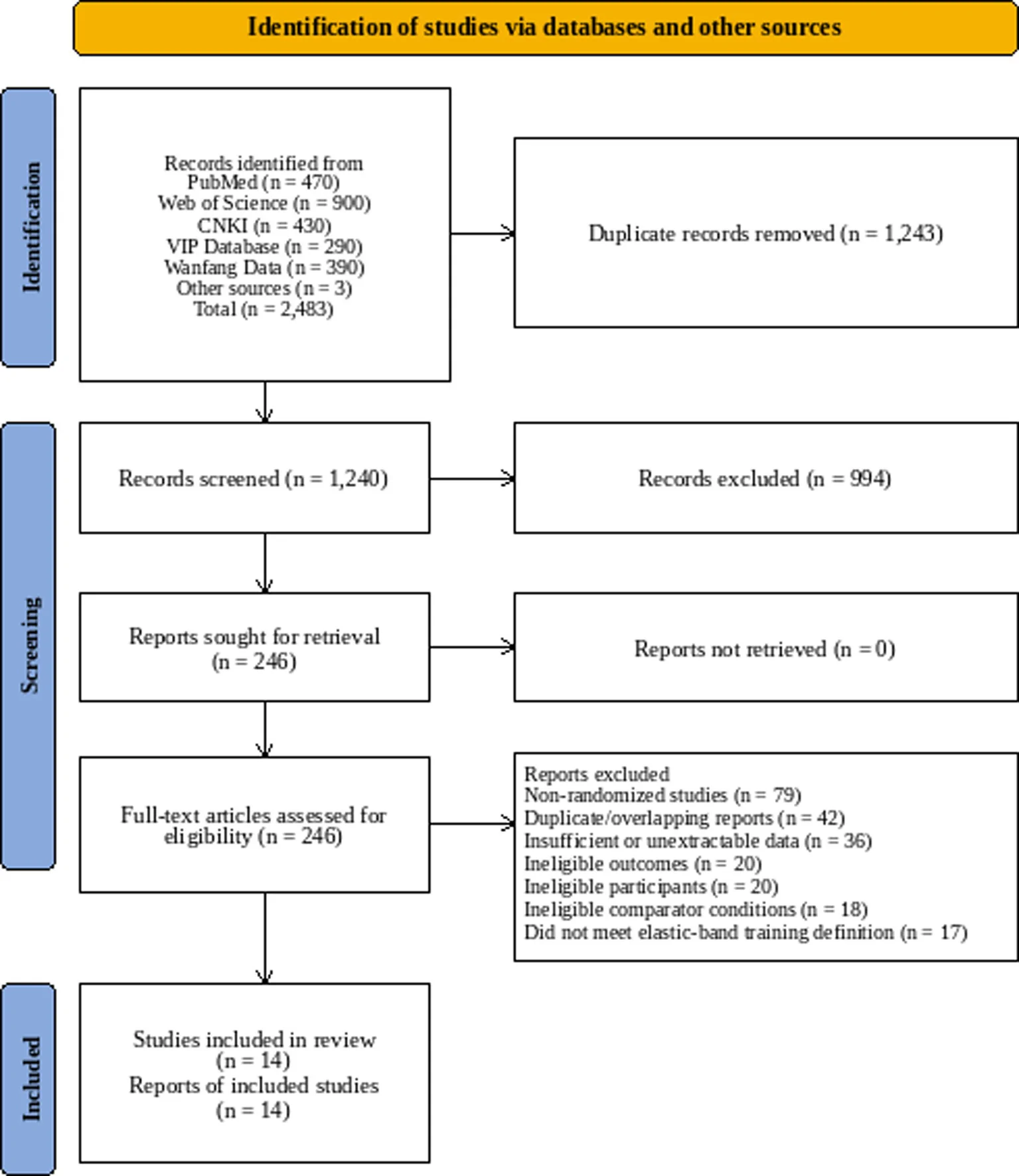
PRISMA 2020 flow diagram of study identification, screening, eligibility assessment, and inclusion.

Study identification and selection followed the PRISMA 2020 process (Figure 1). The five database searches identified 2,480 records: 470 from PubMed, 900 from Web of Science, 430 from CNKI, 290 from VIP Database, and 390 from Wanfang Data. Three additional records were identified from other sources, resulting in 2,483 records. After 1,243 duplicates were removed, 1,240 records underwent title and abstract screening; 994 were excluded at this stage. The remaining 246 full-text reports were assessed for eligibility. Of these, 232 were excluded because they were not randomized controlled trials (*n* = 79), were duplicate publications or reported overlapping data (*n* = 42), had insufficient or unextractable data (*n* = 36), reported ineligible outcomes (*n* = 20), included ineligible participants (*n* = 20), used ineligible comparator conditions (*n* = 18), or did not meet the definition of elastic-band resistance training (*n* = 17). Fourteen studies were included in the systematic review and meta-analysis.

### 3.2 Study characteristics

The 14 included RCTs were published between 2005 and 2025 (15–28) (Table 2). Three were published in 2024 (25–27), two in 2020 (21, 22), two in 2010 (17, 18), and one each in 2025 (28), 2023 (24), 2021 (23), 2012 (20), 2011 (19), 2009 (16), and 2005 (15). The studies included 684 participants, with individual sample sizes ranging from 17 to 100 (15, 26). All participants had T2DM. Several studies focused on clinically defined subgroups, including middle-aged or older adults with sarcopenia (26, 27), prefrailty (24), osteoporosis (25), or knee osteoarthritis (21), as well as women with overweight (17–19). Seven studies used two-arm randomized designs (16, 18, 20, 21, 24–26), and seven used three-arm designs; for the latter, the present meta-analysis extracted data from the elastic-band resistance group and the relevant control group (15, 17, 19, 22, 23, 27, 28). Control conditions mainly involved maintenance of usual lifestyle, standard pharmacological treatment, routine health education, or usual care.

**Table 2:** Characteristics of the included studies.

Study ID	Sample size (E/C included in meta-analysis)	Age/sex profile	Study design	Experimental intervention	Control group	Frequency/duration/intensity	Period	Outcome measures
An et al. (15)	10/7	Total *N* = 26; resistance training 10, aerobic exercise 9, control 7	Three-arm randomized controlled trial; this meta-analysis used resistance training vs. control	Low-intensity circuit resistance training using a resistance-training protocol (including elastic-band/resistance training)	Maintenance of usual lifestyle/control group	5 days/week, twice daily; 15–20RM, approximately 60% 1RM	12 weeks	HbA1c; FPG
Chen et al. (21)	30/30	Dynamic group 65.9 ± 2.9 years; isometric group 65.0 ± 3.1 years	Two-arm randomized controlled trial; dynamic elastic-band training vs. isometric training	Dynamic elastic-band resistance training targeting lower-limb strength and functional performance	Isometric contraction training	3 times/week; about 40 min/session; progressed according to individual capacity	12 weeks	HbA1c; sit-to-stand function
Cheung et al. (16)	20/17	Experimental group 59 ± 8.7 years; control group 62 ± 6.7 years; higher proportion of women	Two-arm parallel randomized controlled trial	Home-based elastic-band resistance training	No additional exercise/usual lifestyle	5 days/week, 30 min/session. Intensity: Dynaband bands with different resistance levels; the appropriate resistance for each exercise was determined in the initial training session; 2 sets × 12 repetitions per exercise; band tension was increased when 12 repetitions could be completed with good technique	16 weeks	Handgrip strength; walking ability
Hu et al. (24)	49/49	98 participants; older inpatients with T2DM and prefrailty	Two-arm randomized controlled trial	Elastic-band resistance training in addition to routine rehabilitation nursing	Routine rehabilitation nursing guidance	3 times/week, 60 min/session. Intensity: early-stage RPE 10–12	12 weeks	HbA1c; FPG; handgrip strength; walking ability; sit-to-stand function; muscle mass
Ku et al. (17)	13/16	Three groups, total *N* = 44; 38–68 years; women	Three-arm randomized controlled trial; this meta-analysis used elastic-band resistance training vs. control	Moderate-intensity elastic-band resistance training	Maintenance of a sedentary lifestyle	5 times/week; moderate-intensity elastic-band training	12 weeks	FPG
Kwon et al. (18)	13/15	Mean age 56.4 ± 7.1 years; diabetes duration 5.9 ± 5.5 years	Two-arm randomized controlled trial	Low-intensity elastic-band resistance training	Usual lifestyle/untrained control	3 days/week; 60 min/session; 40–50% 1RM; 3 sets per exercise	12 weeks	HbA1c; muscle mass
Kwon et al. (19)	12/15	Total *N* = 40; mean age 57.0 ± 6.8 years; BMI 27.0 ± 2.3 kg/m^2^	Three-arm randomized controlled trial; this meta-analysis used elastic-band resistance training vs. control	Elastic-band resistance training	Untrained control; an aerobic exercise group was not used in this comparison	5 days/week; 60 min/day; physical activity monitored by accelerometer	12 weeks	HbA1c
Park et al. (20)	14/13	Experimental group 72.1 ± 4.2 years; control group 69.5 ± 3.4 years	Two-arm randomized controlled trial	Elastic-band resistance training involving multiple upper-limb, trunk, and lower-limb exercises	No participation in other exercise programs/usual lifestyle	3 times/week; 60 min/session; progressed by 10RM/RPE, approximately 50–75% 1RM	12 weeks; plus 8-week follow-up	HbA1c; sit-to-stand function
Ruan et al. (25)	36/38	81 enrolled and 74 completed	Two-arm randomized controlled trial	Resistance-band exercise training program (Thera-Band) in addition to routine treatment and nursing	Routine treatment and nursing care	At least 3 times/week, about 30 min/session. Intensity: moderate; Borg/RPE 12–13; each set of each exercise repeated at least 10 times	12 weeks	HbA1c; FPG
Xia et al. (22)	27/24	77 participants; resistance-only group 81–90 years (84.36 ± 3.54); routine-care group 80–92 years (85.23 ± 5.32)	Three-arm randomized controlled trial; this meta-analysis used elastic-band resistance training alone vs. routine treatment	Elastic-band resistance training (straight-leg deadlift, half squat, standing front leg raise)	Routine diabetes medication treatment	3 times/week, 30 min/session. Intensity: Borg/RPE 12–13, light-to-moderate to moderate intensity	12 weeks	FPG; walking ability; HbA1c removed in sensitivity analysis after the original analysis
Yamamoto et al. (23)	18/17	53 completed; overall mean age 72.9 ± 2.4 years; approximately 47% women	Three-arm randomized controlled trial; this meta-analysis used elastic-band training vs. control	Home-based body-weight plus elastic-band resistance training	Usual management in the control group	7 times/week, 15 min/session. Intensity: 20 repetitions per exercise; THERABAND resistance of 1.3–3.3 kg at 100% elongation; initial resistance selected according to sex and previous exercise habits, with progression to the next resistance level when 20 repetitions were relatively easy	48 weeks	HbA1c; handgrip strength; walking ability; muscle mass
Yan et al. (28)	20/21	75 recruited and 62 completed; elastic-band group 20, control group 21, Tai Chi group 21	Three-arm randomized controlled trial; this meta-analysis used elastic-band group vs. control	Self-designed elastic-band resistance exercise in addition to health education	Maintenance of usual lifestyle plus health education	3 times/week; 40 min/session (5-min warm-up, 30-min training, 5-min cool-down); 12 exercises, 4 circuits, 10–15 repetitions per exercise, 1-min rest between circuits; moderate intensity defined as OMNI-RES 5–6 and target repetitions; 25-lb band for women and 35-lb band for men; resistance reassessed every 2 weeks; heart rate monitored by wearable device; professionally supervised	12 weeks	HbA1c; FPG; handgrip strength; walking ability; sit-to-stand function; muscle mass
Yang et al. (26)	50/50	100 participants; control group 65.77 ± 5.18 years; observation group 65.99 ± 4.78 years	Two-arm randomized controlled trial	Elastic-band resistance exercise in addition to routine exercise guidance	Routine exercise guidance with telephone/WeChat follow-up	2–3 times/week; 30–40 min/session; 3 sets per exercise, 10–15 repetitions; progressed from a yellow elastic band; RPE and %1RM were not reported	24 weeks	HbA1c; FPG
Yao et al. (27)	30/30	PRT, BFRT, and control groups each included 30 participants; each group had 16 men and 14 women	Three-arm randomized controlled trial; this meta-analysis used the PRT group vs. control	Progressive resistance training (PRT) using elastic bands with different resistance levels	Health guidance, routine exercise, T2DM self-management education, and regular follow-up	3 times/week; 30 min/session; resistance progressed from 10 lb. to 15 lb. and then 20 lb	12 weeks	HbA1c; FPG; handgrip strength; walking ability; sit-to-stand function; muscle mass

E/C, experimental/control; T2DM, type 2 diabetes mellitus; HbA1c, glycated hemoglobin; FPG, fasting plasma glucose; RPE, rating of perceived exertion; RM, repetition maximum; PRT, progressive resistance training; BFRT, blood flow restriction training.

Elastic-band resistance training protocols varied substantially in intervention duration, frequency, session length, and technical parameters. Most studies (11 trials) used a 12-week intervention (15, 17–22, 24, 25, 27, 28), whereas the remaining studies evaluated longer interventions lasting 16 weeks (16), 24 weeks (26), or 48 weeks (23). Most protocols prescribed three sessions per week, with each session lasting 30–60 min (18, 20–22, 24, 25, 27, 28). Some studies used high-frequency, low-load programs. For example, An et al. prescribed two sessions per day on 5 days per week (15), whereas Yamamoto et al. prescribed seven 15-min sessions per week (23).

To support safety and effectiveness, several studies used individualized, progressive load adjustment. Intensity was regulated using ratings of perceived exertion (RPE) or repetition maximum (RM) in some studies (20, 22, 24, 25), whereas others progressed resistance by changing band level or repetition number (23, 26, 27). Detailed training parameters are presented in Table 2. All studies assessed outcomes relevant to T2DM management and physical function. HbA1c was reported in 12 studies (15, 18–28), and FPG in eight studies (15, 17, 22, 24–28). Outcomes related to muscle strength and physical function included muscle mass (18, 23, 24, 27, 28), handgrip strength (16, 23, 24, 27, 28), and sit-to-stand function (20, 21, 24, 27, 28). Walking outcomes were reported in six studies (16, 22–24, 27, 28), of which five contributed data suitable for the quantitative synthesis (22–24, 27, 28). Safety reporting was inconsistent: five trials explicitly reported no intervention-related adverse events or serious safety events, one trial described prospective safety monitoring without reporting event results, and eight trials did not report safety outcomes. Study-level safety information is summarized in Supplementary Table S2.

### 3.3 Risk of bias

**Figure 2:**
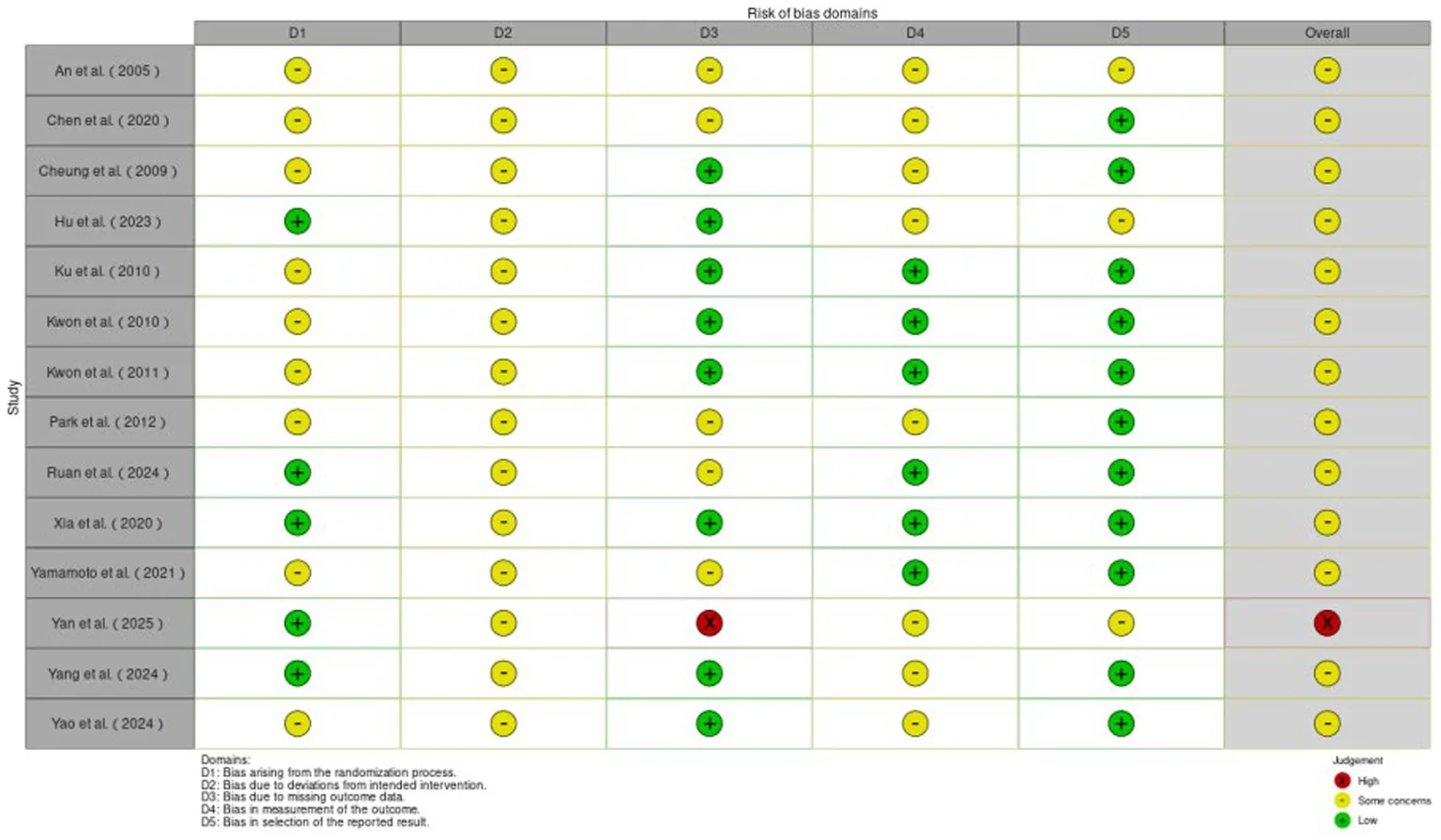
Risk-of-bias summary showing review authors’ judgments for each domain in each included study.

**Figure 3:**
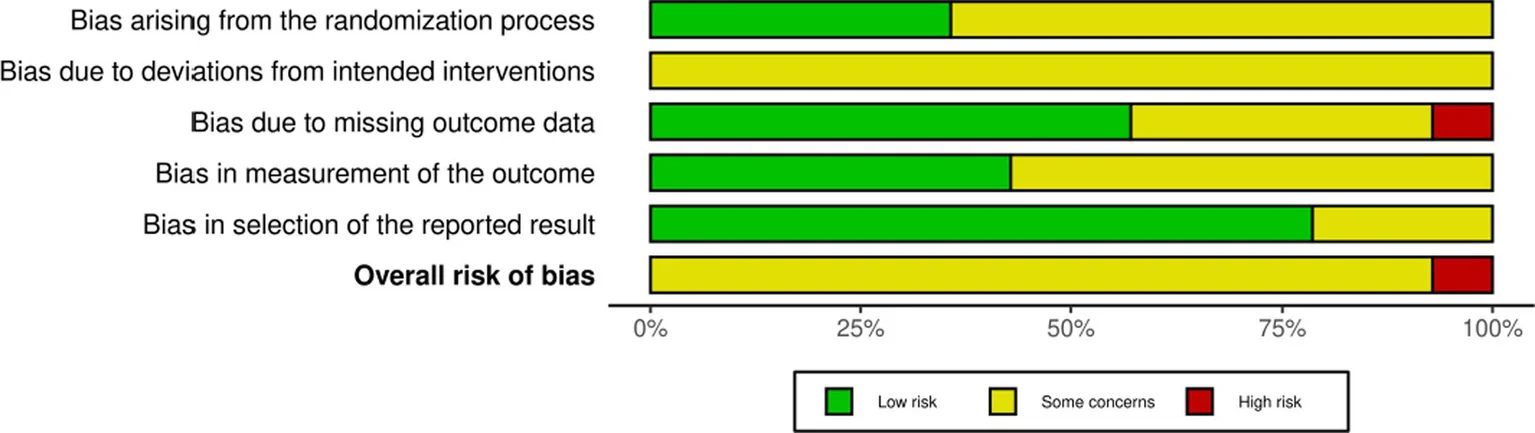
Risk-of-bias graph showing the proportion of included studies rated at each level in the five RoB 2 domains. Assessment methods are described in Section 2.4, and detailed findings are reported in Section 3.3.

The RoB 2 assessment showed that risk-of-bias judgments were concentrated in the “some concerns” category across the 14 included studies (Figures 2, 3). For bias arising from the randomization process (D1), five studies were rated as low risk (22, 24–26, 28) because random sequence generation and allocation concealment were adequately described. The remaining nine studies (15–20, 23, 27) were rated as having some concerns because the methods used to generate the random sequence or conceal allocation were insufficiently reported. For bias due to deviations from intended interventions (D2), all 14 studies (15–28) were rated as having some concerns because participant and provider blinding was not feasible in trials comparing elastic-band exercise with usual lifestyle or usual care. Missing outcome data (D3) was the main source of serious bias. Yan et al. (28) reported substantial or imbalanced missing clinical data without an appropriate method, such as intention-to-treat analysis, and was therefore rated as high risk. An et al. (15), Chen et al. (21), Park et al. (20), and Yamamoto et al. (23) were rated as having some concerns because follow-up data or reasons for attrition were insufficiently reported. The other nine studies (16–19, 22, 24–27) had sufficiently complete data and were rated as low risk. For bias in outcome measurement (D4), six studies reported blinded outcome assessment or used measures that were unlikely to be influenced by assessor awareness and were rated as low risk (17–19, 22, 23, 25). The remaining eight studies (15, 16, 20, 21, 24, 26–28) were rated as having some concerns because assessor blinding was not reported or measurement procedures were not protected against potential bias. For bias in selection of the reported result (D5), 11 studies were rated as low risk (17–23, 25–27). Three studies, An et al. (15), Hu et al. (24), and Yan et al. (28), were rated as having some concerns because prospective trial registration or protocol-based reporting was unavailable. Overall, Yan et al. (28) was rated as high risk because of the serious concerns in D3. The other 13 studies (15–27) were rated as having some concerns, mainly because of the absence of blinding or incomplete reporting of design methods. These limitations were considered when grading the certainty of evidence for glycemic, strength, and functional outcomes.

### 3.4 Meta-analysis results

#### 3.4.1 Glycated hemoglobin (HbA1c)

**Figure 4:**
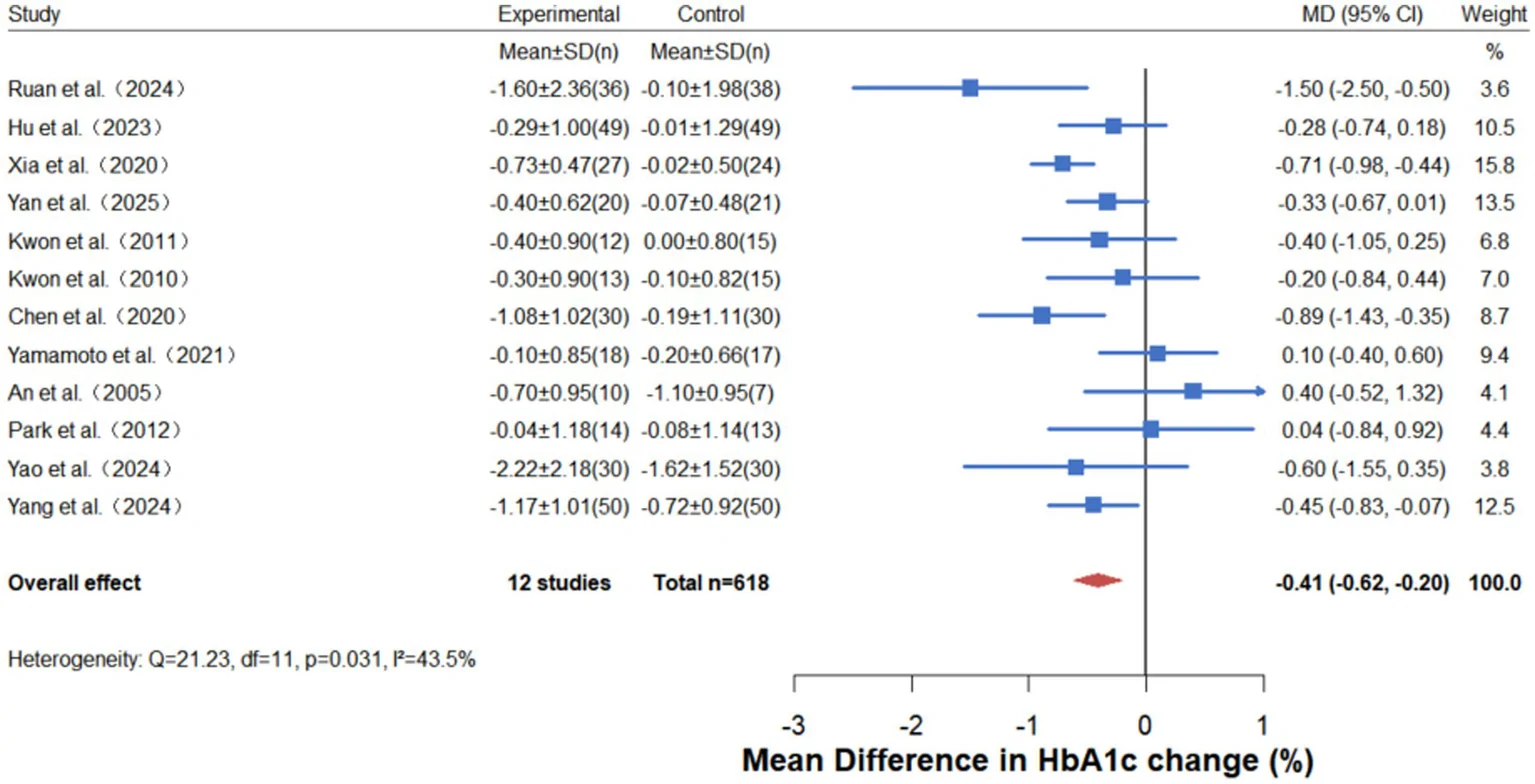
Forest plot of HbA1c before sensitivity analysis.

The meta-analysis suggested that elastic-band resistance training may reduce HbA1c in patients with T2DM. The initial analysis included 12 studies and 618 participants (Figure 4). Compared with the control conditions, elastic-band resistance training produced a statistically significant reduction in HbA1c (MD = −0.41, 95% CI −0.62 to −0.20). Moderate heterogeneity was observed (I^2^ = 43.5%, *p* = 0.031), and a random-effects model was therefore used.

**Figure 5:**
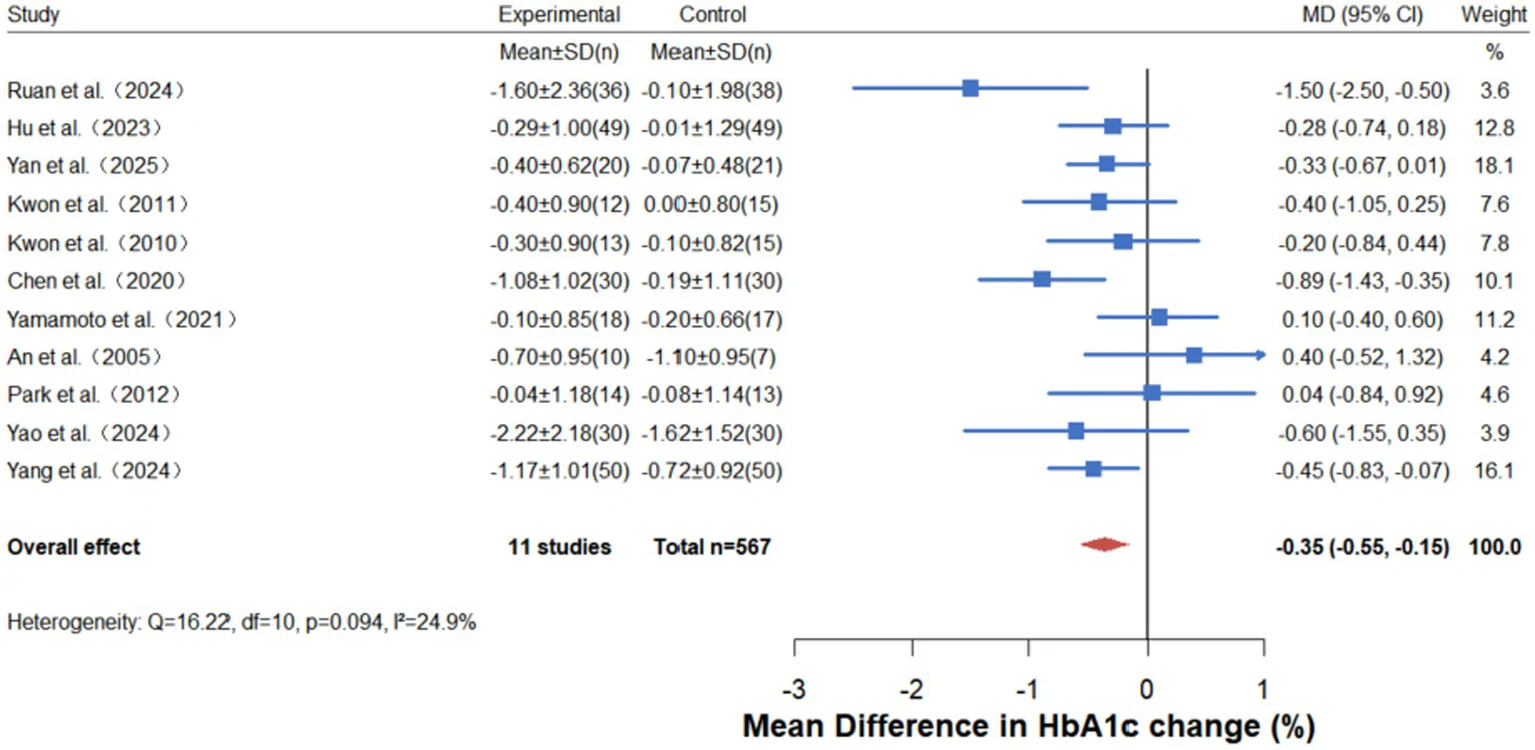
Forest plot of HbA1c after exclusion of Xia et al. (22).

Although the initial heterogeneity was within an acceptable range, a leave-one-out sensitivity analysis was conducted to further examine the robustness of the estimate and identify potential sources of heterogeneity. Xia et al. (22) was identified as the principal contributor (Supplementary Figure 9). After excluding this study, heterogeneity decreased to a low level (I^2^ = 24.9%, *p* = 0.094), and the pooled reduction in HbA1c remained statistically significant (11 studies, *n* = 567; MD = −0.35, 95% CI −0.55 to −0.15; Figure 5). Thus, the direction of effect remained stable after removal of the potential source of heterogeneity. In the sensitivity-analysis dataset, the pooled effect was MD = −0.44 (95% CI −0.62 to −0.25; I^2^ = 27.7%) for training frequencies of ≤3 sessions/week and MD = −0.01 (95% CI −0.40 to 0.38; I^2^ = 14.1%) for >3 sessions/week (P for subgroup difference = 0.0506; Supplementary Figure 2). By comparator type, the pooled effect was MD = −0.23 (95% CI −0.50 to 0.04; I^2^ = 43.7%) against passive controls and MD = −0.51 (95% CI −0.76 to −0.25; I^2^ = 0.2%) against active controls (P for subgroup difference = 0.1451; Supplementary Figure 3). The population-characteristic subgroup test was also not statistically significant (*p* = 0.0934; Supplementary Figure 1). Because several subgroups contained only two to four studies, these interaction tests had limited statistical power; the absence of statistically significant subgroup differences should therefore not be interpreted as evidence of equivalent effects across subgroups.

Leave-one-out sensitivity analysis identified Xia et al. as the principal contributor to between-study heterogeneity. This study enrolled a substantially older population (approximately 80–90 years), implemented a 12-week light-to-moderate elastic-band intervention, and used routine diabetes treatment as the comparator. These characteristics, together with the relatively small sample size and larger between-group change in HbA1c, may explain its influence on the pooled estimate. Nevertheless, the direction and statistical significance of the pooled effect remained unchanged after exclusion of this study, supporting the robustness of the primary findings (22).

#### 3.4.2 Fasting plasma glucose (FPG)

**Figure 6:**
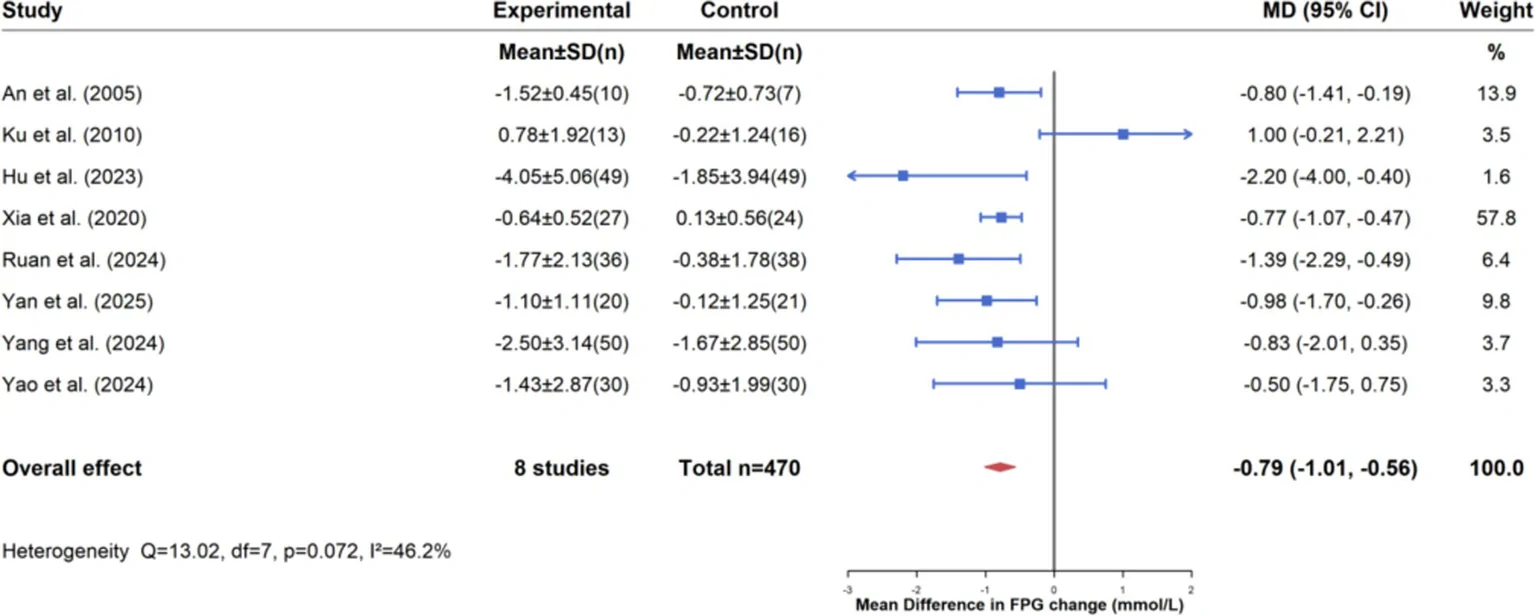
Forest plot of FPG before sensitivity analysis.

Elastic-band resistance training also appeared to improve FPG in patients with T2DM. Eight studies involving 470 participants were pooled (Figure 6). Compared with control conditions, elastic-band resistance training produced a statistically significant reduction in FPG (MD = −0.79, 95% CI −1.01 to −0.56). Some between-study heterogeneity was present (I^2^ = 46.2%, *p* = 0.072). Given the clinical differences in participant characteristics, training protocols, and comparator conditions, a random-effects model was used.

**Figure 7:**
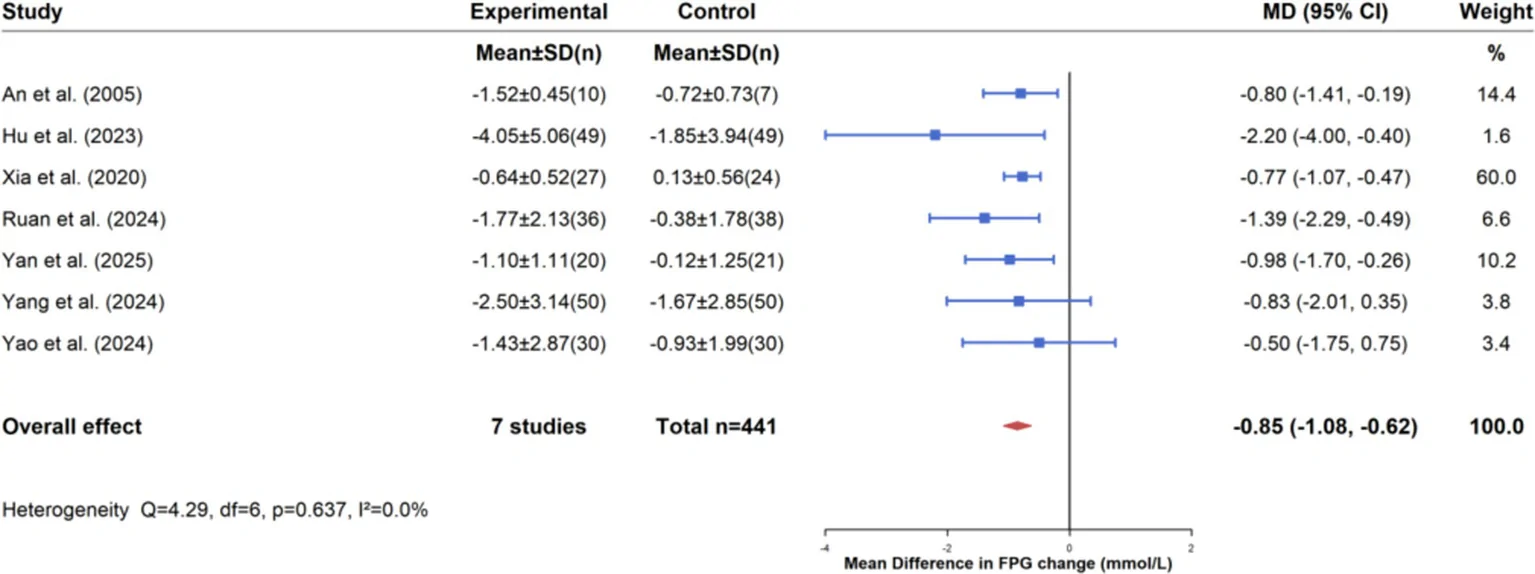
Forest plot of FPG after exclusion of Ku et al. (17).

A leave-one-out sensitivity analysis was conducted to examine robustness and potential sources of heterogeneity. Ku et al. (17) appeared to contribute substantially to heterogeneity (Supplementary Figure 10). After its exclusion, heterogeneity was eliminated (I^2^ = 0.0%, *p* = 0.637), while the pooled effect continued to favor elastic-band training (7 studies, *n* = 441; MD = −0.85, 95% CI −1.08 to −0.62; Figure 7). The direction and statistical significance of the effect were therefore unchanged after removal of the influential study. In subgroup analyses using the full FPG dataset, the pooled effect was MD = −0.86 (95% CI −1.11 to −0.61; I^2^ = 0.0%) for ≤3 sessions/week and MD = 0.02 (95% CI −1.74 to 1.78; I^2^ = 85.4%) for >3 sessions/week (P for subgroup difference = 0.3325; Supplementary Figure 5). By comparator type, the pooled effect was MD = −0.71 (95% CI −1.25 to −0.16; I^2^ = 61.5%) against passive controls and MD = −0.96 (95% CI −1.73 to −0.18; I^2^ = 16.5%) against active controls (P for subgroup difference = 0.6018; Supplementary Figure 6). The population-characteristic subgroup test was not statistically significant (*p* = 0.3671; Supplementary Figure 4). These subgroup analyses were underpowered because of the small numbers of studies, particularly in the >3-sessions/week subgroup, and should be interpreted as exploratory.

Leave-one-out sensitivity analysis identified Ku et al. as the principal contributor to between-study heterogeneity. This study enrolled a relatively younger, female-only population and implemented supervised and home-based elastic-band resistance training five times per week using approximately 40–50% of one-repetition maximum against a sedentary comparator. These differences in participant characteristics, intervention dose, and comparator conditions may explain its influence on the pooled estimate. Exclusion of this study did not alter the direction or statistical significance of the pooled effect, indicating that the overall findings were robust (17).

#### 3.4.3 Secondary outcomes

**Figure 8:**
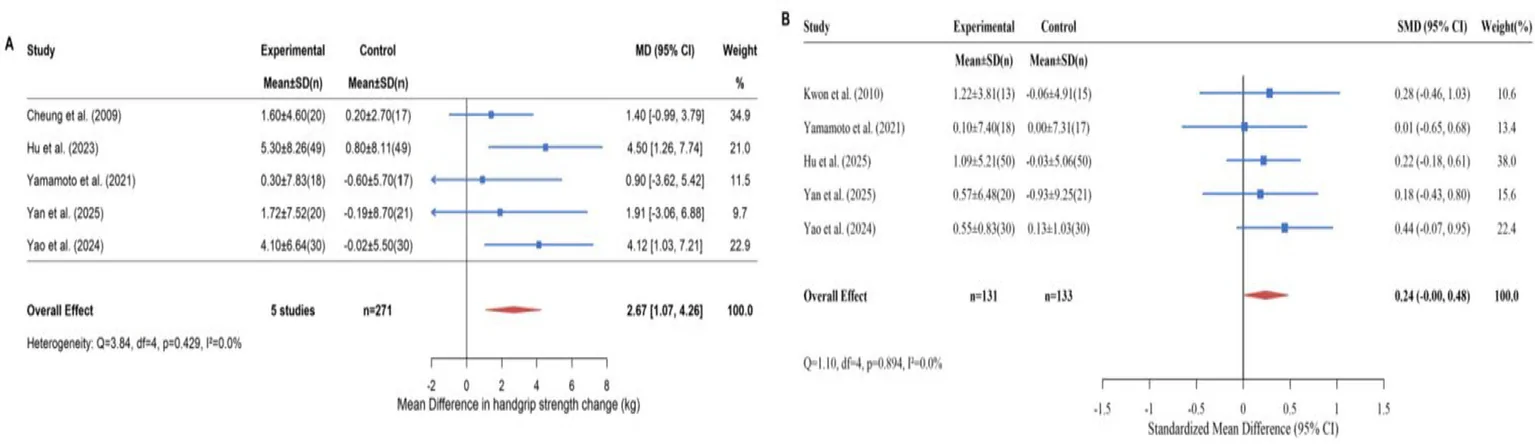
Forest plots of handgrip strength and muscle-mass-related outcomes.

Secondary outcomes included handgrip strength, muscle-mass outcomes, walking outcomes, and sit-to-stand function. Overall, elastic-band resistance training showed relatively consistent favorable trends for muscle strength and muscle mass. The pooled analysis indicated a possible improvement in handgrip strength (5 studies, *n* = 271; MD = 2.67, 95% CI 1.07 to 4.26), with no detectable heterogeneity (I^2^ = 0.0%). Muscle-mass outcomes showed a small favorable point estimate (5 studies, *n* = 264; SMD = 0.24, 95% CI 0.00 to 0.48), with low heterogeneity (I^2^ = 0.0%); however, the small number of studies, measurement diversity, and confidence interval at the null value require cautious interpretation (Figure 8). For physical function, walking ability favored elastic-band training (5 studies, n = 285; SMD = 0.91, 95% CI 0.53 to 1.28), but heterogeneity was moderate (I^2^ = 54%, *p* = 0.07; Supplementary Figure 7). The initial sit-to-stand analysis also favored elastic-band training (5 studies, n = 286; SMD = 2.60, 95% CI 0.69 to 4.51), but heterogeneity was very high (I^2^ = 97%; Supplementary Figure 8a). After exclusion of the influential Hu et al. study, the effect remained favorable (4 studies, *n* = 188; SMD = 1.18, 95% CI 0.36 to 1.99), although substantial heterogeneity persisted (I^2^ = 84.0%; Supplementary Figure 8b). Heterogeneity in walking outcomes likely reflected differences among 4-m and 6-m gait tests, walking time versus speed metrics, baseline functional status, and intervention protocols. For sit-to-stand outcomes, variation between five-repetition and 30-s chair-stand tests, the inclusion of pre-frail inpatients in Hu et al., and differences in co-interventions and baseline functional reserve were plausible sources of heterogeneity. These functional estimates should therefore be interpreted as preliminary overall signals rather than test-specific effects.

The walking analysis combined short-distance gait-performance measures: 6-m walking speed (Xia and Yao), usual gait speed (Yamamoto), 4-m walking speed (Yan), and 6-m walking time (Hu; direction reversed before standardization so that higher values consistently represented improvement). These assessments all quantify usual short-distance gait performance but are reported in different units; SMD was therefore used. Separate meta-analyses by test type were considered but were not feasible because only five studies contributed and most test types were represented by one study. The combined result should consequently be interpreted as an overall gait-performance signal, not as a test-specific effect (22–24, 27, 28).

Muscle-mass outcomes were also heterogeneous in construct and measurement. The contributing studies reported whole-body or regional muscle/lean mass derived from dual-energy X-ray absorptiometry (DXA) and appendicular skeletal muscle mass index (ASMI) derived from bioelectrical impedance analysis. Because these measures are not expressed on a common scale, they were synthesized using SMD. With only five studies, and with most measurement approaches represented by one or two studies, modality-specific meta-analyses were not informative; this measurement diversity is an additional reason to interpret the pooled muscle-mass result cautiously (18, 23, 24, 27, 28).

The sit-to-stand sensitivity analysis is likewise reported as a robustness assessment. Hu et al. enrolled pre-frail older inpatients and delivered elastic-band training in addition to routine rehabilitation, whereas other trials included community-dwelling or clinically different populations and used either five-repetition or 30-s chair-stand tests. These differences in baseline functional reserve, co-intervention context, and outcome scoring may explain the influential result. The high residual heterogeneity after exclusion indicates that the functional estimate remains unstable and should not be regarded as definitive (20, 21, 24, 27, 28).

### 3.5 Publication bias

**Figure 9:**
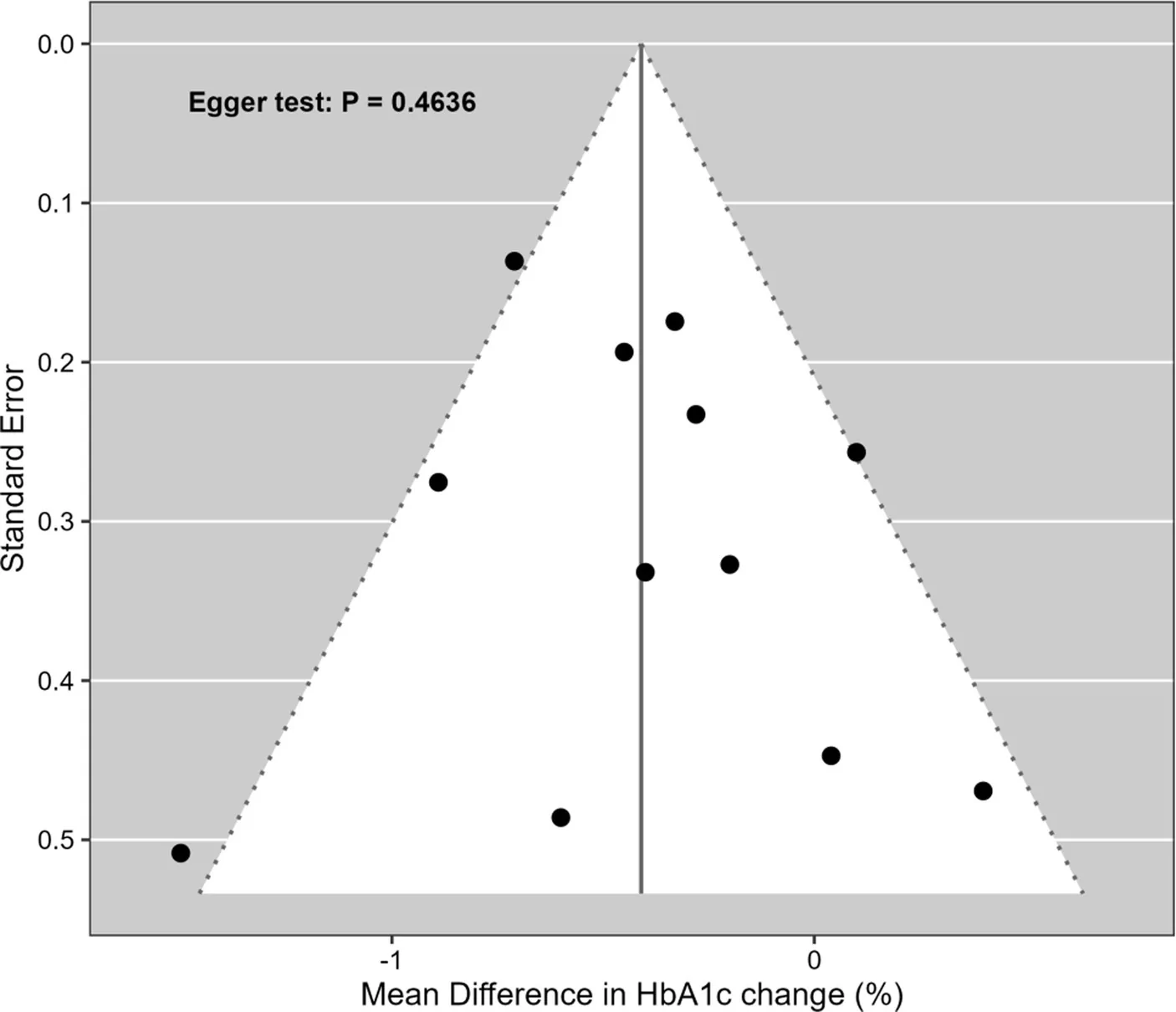
Funnel plot and Egger’s test for publication bias in the HbA1c outcome.

Publication bias for the HbA1c outcome was assessed using a funnel plot and Egger’s test (Figure 9). Mild visual asymmetry was present, and Egger’s test did not detect statistically significant funnel-plot asymmetry (*p* = 0.4636). However, only 12 studies contributed to this analysis, giving the test limited power. Accordingly, the non-significant result should not be interpreted as ruling out publication bias, and small-study effects or unpublished negative findings cannot be excluded. Fewer than 10 studies contributed to each of the FPG, handgrip strength, walking, muscle-mass, and sit-to-stand outcomes; therefore, formal tests were not performed for these outcomes, and publication bias remains uncertain.

### 3.6 Certainty of evidence

Using the GRADE approach, the certainty of evidence ranged from low to moderate across outcomes (Table 3). Certainty was moderate for both primary glycemic outcomes (HbA1c and FPG), with downgrading for risk of bias because several studies had concerns regarding randomization, allocation concealment, blinding, or incomplete outcome data. Certainty was also moderate for handgrip strength because of similar concerns regarding risk of bias. Certainty was low for walking ability, sit-to-stand function, and muscle mass. Walking ability and sit-to-stand function were downgraded for inconsistency because substantial clinical or statistical heterogeneity was present, whereas muscle mass was downgraded for imprecision because only a small number of studies contributed and the confidence interval approached or included the null value (Table 3).

**Table 3:** GRADE assessment of the certainty of evidence.

Outcome	Studies (participants)	Risk of bias	Inconsistency	Indirectness	Imprecision	Publication bias	Certainty of evidence
HbA1c	12 (618)	Downgraded by 1: RoB 2 concerns	Not downgraded	Not downgraded	Not downgraded	Not downgraded: Egger *p* = 0.4636; low power	⊕ ⊕ ⊕⊝ Moderate
FPG	8 (470)	Downgraded by 1: RoB 2 concerns	Not downgraded	Not downgraded	Not downgraded	Not assessed: <10 studies; cannot exclude	⊕ ⊕ ⊕⊝ Moderate
Handgrip strength	5 (271)	Downgraded by 1: RoB 2 concerns	Not downgraded	Not downgraded	Not downgraded	Not assessed: <10 studies; cannot exclude	⊕ ⊕ ⊕⊝ Moderate
Walking ability	5 (285)	Downgraded by 1: RoB 2 concerns	Downgraded by 1: I^2^ = 54%	Not downgraded	Not downgraded	Not assessed: <10 studies; cannot exclude	⊕ ⊕ ⊝⊝ Low
Sit-to-stand function	5 (286)	Downgraded by 1: RoB 2 concerns	Downgraded by 1: substantial heterogeneity	Not downgraded	Not downgraded	Not assessed: <10 studies; cannot exclude	⊕ ⊕ ⊝⊝ Low
Muscle mass	5 (264)	Downgraded by 1: RoB 2 concerns	Not downgraded	Not downgraded	Downgraded by 1: CI near/null; n = 264	Not assessed: <10 studies; cannot exclude	⊕ ⊕ ⊝⊝ Low

Certainty of evidence was assessed using the GRADE approach. HbA1c, glycated hemoglobin; FPG, fasting plasma glucose; CI, confidence interval.

GRADE downgrade explanations: risk of bias was downgraded by one level for every outcome because most trials had RoB 2 “some concerns,” most commonly incomplete reporting of randomization or allocation concealment, unavoidable lack of participant/provider blinding in exercise trials, and/or incomplete outcome data. Inconsistency was downgraded by one level for walking ability (I^2^ = 54%) and sit-to-stand function because heterogeneity remained substantial and clinically plausible across the relevant analyses. Imprecision was downgraded by one level for muscle mass because only five small studies contributed and the 95% CI approached or included the null. Indirectness was not downgraded because the populations, intervention, comparator, and outcomes addressed the review question. Publication bias was not formally assessable for outcomes with fewer than 10 studies and could not be excluded.

## 4 Discussion

### 4.1 Summary of findings

This systematic review and meta-analysis evaluated the effects of elastic-band resistance training on glycemic control, muscle strength, and physical function in patients with T2DM. Based on 14 RCTs involving 684 participants, the primary analyses including all eligible studies showed reductions in HbA1c (MD = −0.41, 95% CI −0.62 to −0.20) and FPG (MD = −0.79, 95% CI −1.01 to −0.56), with moderate certainty of evidence for both outcomes. Leave-one-out sensitivity analyses yielded similar effect directions and statistical conclusions, supporting the robustness of the primary findings without replacing the primary estimates. Handgrip strength improved consistently (MD = 2.67, 95% CI 1.07 to 4.26; I^2^ = 0.0%), with moderate-certainty evidence. Muscle-mass outcomes showed a small favorable point estimate (SMD = 0.24, 95% CI 0.00 to 0.48), but certainty was low. Walking ability also showed a favorable pooled estimate (SMD = 0.91), although heterogeneity was moderate (I^2^ = 54%). A sit-to-stand sensitivity analysis favored elastic-band training, but residual heterogeneity remained high (I^2^ = 84%), limiting the stability of that estimate. Collectively, the findings suggest that elastic-band resistance training may offer a feasible adjunctive strategy for glycemic control and muscle strength, while functional and muscle-mass findings remain preliminary.

The primary HbA1c reduction of 0.41 percentage points was modest but potentially clinically relevant. Its magnitude is broadly consistent with reductions reported for structured exercise and resistance-training interventions in previous meta-analyses (5, 29). Current American Diabetes Association guidance emphasizes individualized glycemic targets and recommends resistance training as tolerated for older adults who can participate safely (29). Because no universal exercise-specific minimal important difference for HbA1c has been established, the clinical importance of this average effect will depend on baseline glycemic status, medication use, comorbidities, treatment goals, and durability. Elastic-band resistance training should therefore be framed as an adjunct to, rather than a substitute for, glucose-lowering medication, dietary management, and individualized diabetes care.

The pooled handgrip-strength improvement of 2.67 kg was measurable but did not reach the 5.0–6.5 kg range proposed as a reasonable estimate of meaningful change in a systematic review; published thresholds vary by population, and no T2DM-specific threshold has been established (30). Moreover, Asian Working Group for Sarcopenia cutoffs of <28 kg for men and <18 kg for women are diagnostic thresholds for low strength rather than thresholds for longitudinal change (31). The present result should therefore not be described as definitively clinically important, and the aggregate data do not show how many participants crossed a sarcopenia threshold or experienced clinically meaningful functional recovery.

Pooling usual care, education, rehabilitation, and active-exercise comparators yields an average incremental effect across different clinical contrasts rather than a single uniform treatment effect. Although smaller effects might theoretically be expected against active exercise, this pattern was not observed consistently: the active-control subgroup retained favorable point estimates for both HbA1c and FPG, and the tests for subgroup differences were not statistically significant. These findings should not be interpreted as evidence that comparator intensity is unimportant, because active controls included clinically different interventions, only three to four studies contributed to these subgroups, and comparator type was potentially confounded by population characteristics and co-interventions. The pooled estimates should therefore be interpreted as average effects across heterogeneous care contexts rather than as proof of superiority over any specific active exercise modality.

### 4.2 Comparison with previous evidence and potential mechanisms

Our findings are consistent with the broader literature on exercise interventions for T2DM while specifically addressing elastic-band training. An updated meta-analysis of 46 RCTs involving 2,130 patients found that resistance training reduced HbA1c (MD = −0.50, 95% CI −0.67 to −0.34) and fasting glucose (32). The reduction in HbA1c observed in the present review (MD = −0.41) was directionally consistent but somewhat smaller, possibly because elastic bands generally provide lower loads and facilitate more independent training, whereas the previous review included machine-based and free-weight programs. A meta-analysis comparing home-based and gym-based resistance training in patients with T2DM found a significant HbA1c reduction after gym-based training but not after home-based training; the authors attributed the difference to limited supervision, imprecise loading, and reduced adherence in home settings (33). Most elastic-band interventions in our review retained some degree of instruction or supervision and produced statistically significant glycemic improvements. This suggests that accessible home-based elastic-band training may still improve glycemic control when appropriate instruction and progression are provided. The favorable findings for muscle strength and physical function are also consistent with previous evidence on elastic-band training in older adults (34), and extend that evidence to patients with T2DM through an integrated assessment of glycemic control, strength, muscle mass, and physical function.

The present meta-analysis did not directly measure insulin sensitivity, GLUT4 expression, muscle protein synthesis, or neuromuscular adaptation. The following explanations are therefore based on previous research and physiological reasoning and should not be interpreted as mechanisms directly demonstrated by this review (35). Skeletal muscle is a major site of insulin-stimulated peripheral glucose uptake and disposal and plays a central role in whole-body glucose homeostasis (36). Exercise-induced muscle contraction can promote GLUT4 translocation to the cell membrane and increase skeletal muscle glucose uptake; chronic training may also increase GLUT4 expression and glycogen storage capacity (37). Mechanistic studies in patients with T2DM have shown that strength training can increase insulin-mediated skeletal muscle glucose uptake, GLUT4 content, and the expression of insulin-signaling proteins (38). Thus, the observed improvements in HbA1c and FPG may be related to enhanced skeletal muscle glucose uptake, improved insulin action, and greater glycogen synthesis capacity, although these pathways were not directly measured in the included studies. Resistance training may also indirectly reduce insulin resistance by improving abdominal adiposity, fasting glucose, insulin sensitivity, and selected inflammatory markers (39, 40); however, most included trials did not report visceral fat, lipid profiles, inflammatory markers, or direct measures of insulin sensitivity, so these effects should not be presented as findings of the present review.

At the structural and functional level, regular resistance training stimulates muscle protein synthesis and, over time, supports muscle remodeling and hypertrophic adaptation (41). Maintaining or increasing skeletal muscle mass provides a structural basis for daily activities, walking, and sit-to-stand transfers. Because patients with T2DM, particularly older adults, are at risk of reduced muscle strength and mass, the favorable changes in handgrip strength and muscle mass observed in this review suggest that elastic-band training may also help counter functional decline (42). Early strength gains during resistance training can precede detectable hypertrophy and may reflect increased motor-unit recruitment, enhanced neural drive, and improved neuromuscular coordination (43). The absence of heterogeneity in the handgrip-strength analysis (I^2^ = 0.0%) suggests a consistent direction of effect, but the limited number of studies still requires caution.

Because elastic-band resistance is adjustable, versatile, portable, and suitable for home use, it can be incorporated into functional exercises related to chair transfers and walking and may theoretically improve these activities. Nevertheless, heterogeneity was high for several functional outcomes, and the estimates were not stable (44). Nonsignificant or inconsistent functional findings may reflect short intervention durations, variation in training prescriptions and measurement tools, and the small evidence base. Previous dose–response research also indicates that training duration, frequency, intensity, and volume can influence the magnitude of strength and morphological adaptation (45). Future studies should use more standardized prescriptions, provide longer follow-up, and assess mechanistic biomarkers alongside clinical outcomes to clarify how elastic-band resistance training affects glycemic control, muscle strength, and physical function.

### 4.3 Strengths and limitations

This review has several methodological strengths. It included only RCTs, used a prospectively registered protocol, integrated multiple clinically relevant outcomes, and conducted sensitivity analyses, subgroup analyses, publication bias assessment, and GRADE evaluation. Several limitations must nevertheless be considered. First, the number of studies and total sample size were relatively small: 14 RCTs included 684 participants, and only four or five studies contributed to several secondary outcomes. Statistical power was therefore limited, some confidence intervals were wide or crossed the null, and the generalizability of the estimates was restricted. Second, the interventions were clinically heterogeneous, with substantial differences in training duration, frequency, intensity regulation, and exercise content. Reporting of training prescriptions was also inconsistent; several trials did not provide quantitative intensity metrics such as RPE, band tension, or percentage of 1RM, limiting replication and dose–response interpretation. Although this diversity reflects real-world elastic-band practice, it complicates identification of an optimal prescription and may explain the heterogeneity in walking ability (I^2^ = 54%) and sit-to-stand function (I^2^ = 84%). Third, long-term follow-up data were largely absent. Of the 14 studies, only Park et al. reported outcomes eight weeks after the intervention; all other studies ended assessment at the postintervention timepoint. Evidence is therefore insufficient to determine whether the benefits of elastic-band training are maintained over time.

Safety reporting requires particular caution. Five trials explicitly reported no intervention-related adverse events or serious safety events, one described prospective monitoring without presenting event results, and the remaining eight did not report safety outcomes. Consequently, the absence of reported events cannot be interpreted as evidence that unsupervised home-based elastic-band training is uniformly safe. In older or frail adults with T2DM, initial professional assessment and instruction, gradual progression of band resistance, stable support during standing exercises, and monitoring for hypoglycemia, musculoskeletal pain, falls, and exercise intolerance are advisable. Additional precautions may be required for patients with peripheral or autonomic neuropathy, active foot lesions, severe balance impairment, proliferative retinopathy, unstable cardiovascular disease, or painful joint disorders. The available trials did not permit quantitative estimation of these harms, and systematic adverse-event definitions and reporting should be mandatory in future studies (Supplementary Table S2).

Additional limitations are the unavoidable absence of participant and provider blinding in exercise trials, variable elastic-band prescriptions, and clinically diverse control conditions. These issues may have introduced performance effects and affected the magnitude of pooled estimates. Most studies were conducted in Asian populations, so the generalizability of the findings to other regions, health systems, and ethnic groups is uncertain. Together with the small number of RCTs, this supports cautious, rather than definitive, conclusions, particularly for walking ability, sit-to-stand function, and muscle mass.

### 4.4 Implications for practice and research

Despite the moderate-to-low certainty of the evidence, the findings may inform clinical practice. Elastic-band resistance training may be considered as an adjunct to comprehensive exercise management for patients with T2DM, based particularly on the moderate-certainty evidence for improvements in glycemic control and handgrip strength. This approach may be especially useful for patients who cannot sustain conventional machine-based training because of financial constraints, mobility limitations, or lack of access to specialized facilities. When developing individualized prescriptions, clinicians may consider the protocol characteristics most commonly studied in the included trials—moderate-intensity training approximately three times per week for 30–60 min per session and for at least 12 weeks—while recognizing that these parameters do not represent an established optimal prescription. Professional instruction should be provided during the initial stage to ensure safe technique and appropriate loading. For older patients with sarcopenia, frailty, or joint disorders, the low-impact and progressively adjustable nature of elastic-band resistance may be better tolerated than free weights. Training may begin with functional movements and progress according to periodic assessment. However, subgroup analyses did not identify significant differences by training frequency, population characteristics, or comparator type. The current evidence is therefore insufficient to support distinct recommendations for specific subgroups, and clinical decisions should be individualized.

From a public health and policy perspective, these findings provide a preliminary rationale for incorporating elastic-band resistance training into community-based exercise management for T2DM. Low-cost, home-accessible resistance tools could be included among resources recommended in diabetes self-management education, accompanied by appropriate investment in community implementation and training for health professionals. Future research should prioritize adequately powered, multicenter RCTs with follow-up durations of at least 6 months; systematically evaluate dose–response relationships across training duration, frequency, intensity, and volume; and embed standardized adverse-event monitoring in trial protocols. Development and adoption of a core outcome set, particularly for muscle mass, gait performance, and sit-to-stand function, would reduce measurement heterogeneity and improve comparability across trials. Future studies should also systematically control and document key confounders, including changes in glucose-lowering medication, dietary management, and habitual physical activity, to improve the reliability of effect estimates.

Integration with digital health technologies also warrants investigation. Combining wearable devices, mobile applications, and remote supervision with elastic-band training could support real-time monitoring of adherence, training load, movement quality, glucose responses, and early safety signals. These technologies may help standardize home-based delivery and permit timely adjustment of resistance or exercise technique. However, adequately powered trials are needed to determine whether digitally supported elastic-band programmes improve adherence, safety, glycemic outcomes, and functional recovery compared with conventional instruction alone.

## 5 Conclusion

Evidence from 14 RCTs involving 684 participants suggests that elastic-band resistance training may produce modest improvements in glycemic control in patients with T2DM. In the primary analyses, HbA1c decreased by 0.41 percentage points (MD = −0.41, 95% CI −0.62 to −0.20) and FPG by 0.79 mmol/L (MD = −0.79, 95% CI −1.01 to −0.56), with moderate certainty of evidence. Handgrip strength also improved consistently (MD = 2.67, 95% CI 1.07 to 4.26), supported by moderate-certainty evidence. Evidence regarding skeletal muscle mass, walking ability, and sit-to-stand function remains uncertain because of the small number of trials, heterogeneous measurements or comparator conditions, and low certainty for these outcomes. Because it is inexpensive, portable, and suitable for home use, elastic-band resistance training may be considered as an adjunct to comprehensive exercise management for T2DM, particularly in community and home settings, but not as a replacement for pharmacological or dietary management. Safety remains insufficiently characterized because adverse events were incompletely reported. Larger, adequately powered multicenter RCTs with follow-up of at least 6 months, standardized outcome measures, and systematic safety reporting are needed to determine the optimal training prescription and durability of effects.

## Data Availability

The original contributions presented in the study are included in the article/Supplementary material, further inquiries can be directed to the corresponding author.

## Glossary

T2DM: Type 2 diabetes mellitus
HbA1c: Glycated hemoglobin
FPG: Fasting plasma glucose
RCT: Randomized controlled trial
PICO-S: Population, Intervention, Comparator, Outcome, Study design
PRISMA: Preferred Reporting Items for Systematic Reviews and Meta-Analyses
PROSPERO: International Prospective Register of Systematic Reviews
MeSH: Medical Subject Headings
CNKI: China National Knowledge Infrastructure
VIP: VIP Database (Chinese scientific journals database)
RoB 2: Cochrane Risk of Bias 2 tool
GRADE: Grading of Recommendations Assessment, Development and Evaluation
MD: Mean difference
SMD: Standardized mean difference
CI: Confidence interval
RPE: Rating of perceived exertion
RM: Repetition maximum
1RM: One-repetition maximum
PRT: Progressive resistance training
BFRT: Blood flow restriction training
BMI: Body mass index
ITT: Intention-to-treat
ADA: American Diabetes Association
GLUT4: Glucose transporter type 4
ADL: Activities of daily living
