# Supplementary material for: Effects of elastic-band resistance training on glycemic control, muscle strength, and functional outcomes in patients with type 2 diabetes mellitus: a systematic review and meta-analysis of randomized controlled trials
Source: Front Public Health. 2026 Aug 21;14:1910792. doi: 10.3389/fpubh.2026.1910792 (PMC13541539; doi:10.3389/fpubh.2026.1910792)
Supplement: Supplementary file 1 [file Supplementary_file_1.docx]

**Supplementary Material**

**Supplementary Table S1. Complete search strategies**

Databases were searched from inception to June 5, 2026. No language or publication-date restrictions were applied in the search strings shown below.

| **Database** | **Complete search strategy** |
| --- | --- |
| **PubMed** | #1 "Diabetes Mellitus, Type 2"[Mesh] #2 ("type 2 diabetes" OR "type 2 diabetes mellitus" OR T2DM OR "non-insulin-dependent diabetes" OR "diabetes mellitus type 2")[Title/Abstract] #3 "Resistance Training"[Mesh] OR "Exercise Therapy"[Mesh] OR "Elastic Bandages"[Mesh] #4 ("elastic band*" OR "elastic bands" OR "resistance band*" OR "Thera-Band" OR Theraband OR "elastic tubing" OR "elastic resistance" OR "rubber band*" OR "progressive elastic resistance" OR "elastic resistance training" OR "elastic band resistance exercise" OR "elastic resistance exercise" OR "elastic tubing exercise" OR "home resistance exercise")[Title/Abstract] #5 "Randomized Controlled Trial"[Publication Type] OR "Controlled Clinical Trial"[Publication Type] #6 (randomized OR randomised OR randomly OR "random allocation" OR "controlled trial" OR "clinical trial" OR RCT)[Title/Abstract] #7 (#1 OR #2) AND (#3 OR #4) AND (#5 OR #6) |
| **Web of Science** | TS=("type 2 diabetes" OR "type 2 diabetes mellitus" OR T2DM OR "non-insulin-dependent diabetes") AND TS=("elastic band*" OR "resistance band*" OR "Thera-Band" OR Theraband OR "elastic tubing" OR "elastic resistance" OR "elastic resistance training" OR "elastic resistance exercise" OR "elastic tubing exercise" OR "home resistance exercise") AND TS=(randomized OR randomised OR randomly OR "controlled trial" OR RCT) |
| **CNKI** | SU=('2型糖尿病' OR '2型糖尿病患者') AND SU=('弹力带' OR '弹力带训练' OR '弹性阻力训练' OR '抗阻训练') AND SU=('随机对照试验' OR '随机' OR 'RCT') |
| **VIP** | SU=('2型糖尿病' OR '2型糖尿病患者') AND SU=('弹力带' OR '弹力带训练' OR '弹性阻力训练' OR '抗阻训练') AND SU=('随机对照试验' OR '随机' OR 'RCT') |
| **Wanfang** | SU=('2型糖尿病' OR '2型糖尿病患者') AND SU=('弹力带' OR '弹力带训练' OR '弹性阻力训练' OR '抗阻训练') AND SU=('随机对照试验' OR '随机' OR 'RCT') |

Abbreviations: CNKI, China National Knowledge Infrastructure; RCT, randomized controlled trial; T2DM, type 2 diabetes mellitus; VIP, VIP Database; Wanfang, Wanfang Data.

**Supplementary Table S2. Adverse-event and safety reporting in the included studies**

| **Study** | **Adverse-event and safety reporting** |
| --- | --- |
| An et al. (2005) | Adverse events were not reported. |
| Chen et al. (2020) | The report stated that no hypoglycemia or other exercise-related adverse events occurred; 10 participants did not complete follow-up. |
| Cheung et al. (2009) | No training-related adverse events were reported. Three participants withdrew, and medication changes were documented in seven participants. |
| Hu et al. (2023) | Prospective safety monitoring was described, but adverse-event results were not reported. |
| Ku et al. (2010) | Adverse events were not reported. |
| Kwon et al. (2010) | Adverse events were not reported. |
| Kwon et al. (2011) | Adverse events were not reported. |
| Park et al. (2012) | Adverse events were not reported; participant numbers decreased from 15/15 at enrollment to 14/13 at analysis. |
| Ruan et al. (2024) | No exercise-related adverse events were reported. Six participants withdrew for reasons including hot weather or household work. |
| Xia et al. (2020) | Adverse events were not reported. |
| Yamamoto et al. (2021) | No exercise- or nutrition-related adverse events were reported. Seven participants withdrew, with reasons described in the report. |
| Yan et al. (2025) | No exercise-related adverse events were reported. Thirteen participants did not complete the trial. |
| Yang et al. (2024) | Adverse events were not reported. |
| Yao et al. (2024) | No hypoglycemia or falls were reported in the progressive-resistance-training and control groups included in this meta-analysis. One bruise occurred in the blood-flow-restriction group, which was not included in the pooled comparison. |

Note. “Not reported” indicates that the source report did not provide a systematic adverse-event statement; it should not be interpreted as evidence that no adverse events occurred.

Supplementary Figure 1. Subgroup analysis of HbA1c by clinical characteristics

Supplementary Figure 2. Subgroup analysis of HbA1c by training frequency

Supplementary Figure 3. Subgroup analysis of HbA1c by comparator type

Supplementary Figure 4. Subgroup analysis of FPG by clinical characteristics

Supplementary Figure 5. Subgroup analysis of FPG by training frequency

Supplementary Figure 6. Subgroup analysis of FPG by comparator type

Supplementary Figure 7. Forest plot of walking ability-related outcomes

Supplementary Figure 8a. Forest plot of sit-to-stand-related outcomes before excluding Hu et al.

Supplementary Figure 8b. Forest plot of sit-to-stand-related outcomes after excluding Hu et al.

Supplementary Figure 9. Leave-one-out sensitivity analysis of HbA1c

Supplementary Figure 10. Leave-one-out sensitivity analysis of FPG
